# Generation of multiphoton quantum states on silicon

**DOI:** 10.1038/s41377-019-0153-y

**Published:** 2019-05-01

**Authors:** Ming Zhang, Lan-Tian Feng, Zhi-Yuan Zhou, Yang Chen, Hao Wu, Ming Li, Shi-Ming Gao, Guo-Ping Guo, Guang-Can Guo, Dao-Xin Dai, Xi-Feng Ren

**Affiliations:** 1 State Key Laboratory for Modern Optical Instrumentation, Centre for Optical and Electromagnetic Research, Zhejiang Provincial Key Laboratory for Sensing Technologies, College of Optical Science and Engineering, Zhejiang University, Zijingang Campus, Hangzhou, 310058 China; 2 Ningbo Research Institute, Zhejiang University, Ningbo, 315100 China; 3 Key Laboratory of Quantum Information, CAS, University of Science and Technology of China, Hefei, Anhui 230026 China; 4 Synergetic Innovation Center of Quantum Information & Quantum Physics, University of Science and Technology of China, Hefei, Anhui 230026 China

**Keywords:** Quantum optics, Integrated optics, Silicon photonics

## Abstract

Multiphoton quantum states play a critical role in emerging quantum technologies and greatly improve our fundamental understanding of the quantum world. Integrated photonics is well recognized as an attractive technology offering great promise for the generation of photonic quantum states with high-brightness, tunability, stability, and scalability. Herein, we demonstrate the generation of multiphoton quantum states using a single-silicon nanophotonic waveguide. The detected four-photon rate reaches 0.34 Hz even with a low-pump power of 600 μW. This multiphoton quantum state is also qualified with multiphoton quantum interference, as well as quantum state tomography. For the generated four-photon states, the quantum interference visibilities are greater than 95%, and the fidelity is 0.78 ± 0.02. Furthermore, such a multiphoton quantum source is fully compatible with the on-chip processes of quantum manipulation, as well as quantum detection, which is helpful for the realization of large-scale quantum photonic integrated circuits (QPICs) and shows great potential for research in the area of multiphoton quantum science.

## Photonics: Pairing-up for next generation photonic quantum technologies

Chinese scientists have developed a technique for generating photon-pairs for use in quantum devices, paving the way for a range of new photonic quantum technologies. Multi-photon quantum sources are critical for the development of new photonic quantum technologies, and drove Dao-Xin Dai and colleagues from Zhejiang University, in collaboration with researchers from the University of Science and Technology of China, to develop a technique that generates high-quality photonic quantum states. Using a method called spontaneous four-wave mixing, whereby three electromagnetic fields interact to produce a fourth field, the team created multi-photon quantum states in a silicon nanophotonic spiral waveguide. The technique produces bright, tunable, stable and scalable multi-photon quantum states, and is compatible with a current fiber and integrated circuit manufacturing processes, opening the door to new photonic quantum technologies in communications, computation, and imaging.

## Introduction

Multiphoton quantum sources are critical resources^1^ for quantum communication^2^, computation^3,4^, simulation^5^, and metrology^6^. Great efforts have been made for the realization of high-quality, bright, and scalable multiphoton quantum states to enable powerful implementations of quantum technologies. One of the promising methods to generate multiphoton quantum sources is to multiplex several biphoton sources with the assistance of postselections^7–9^. On the other hand, the efficiency of the multiplexing process decreases exponentially with the number of entangled photons; thus, it is essential to achieve bright biphoton sources with high fidelity^9^. In comparison with free-space optics, integrated photonics has been recognized as a preferred platform for realizing quantum photon-pair sources^10–17^, regarding the compatibility with chip-based processes of quantum manipulation and detection^18–21^. Furthermore, the strong mode confinement in optical waveguides and the filed enhancement in high-Q optical cavities can greatly enhance the nonlinear optical interactions, so that it is possible to efficiently achieve on-chip multiphoton quantum sources. For example, chip-based multiple indistinguishable heralded single-photon source preparation^22,23^ and the generation of multiphoton quantum state with time-bin encoding^24^ have been reported.

In various platforms for quantum photonic integrated circuits (QPICs), silicon-on-insulator (SOI) technology has been considered to be one of the most promising options for realizing high-quality photon-pair sources because of its unique advantages. First, silicon has high third-order optical nonlinearity, which is at least one order of magnitude greater than that of glass. Second, an SOI nanophotonic waveguide has an ultrahigh refraction index contrast, which enables very strong nonlinear interactions as desired. Third, silicon photonics has complementary metal–oxide–semiconductor compatibility, which is very attractive for large-scale photonic integration^25^. With these advantages, high-quality silicon-based on-chip biphoton quantum sources can be realized (see Supplementary Table S1) and further used for quantum information processings^10,26–32^. However, generation of multiphoton quantum states on silicon has not yet been seriously considered or reported.

In this paper, we demonstrate the generation of four-photon polarization-encoding quantum states via the degenerated spontaneous four-wave mixing (SFWM) process in a silicon spiral waveguide. Herein, we first demonstrate the biphoton Bell entangled quantum states with high brightness (270 kHz) with a high coincidence to accidental ratio (CAR) (~230) at a low-pump power (120 μW). The four-photon quantum state is then realized through the tensor product of two biphoton Bell entangled states. The detected four-photon rate achieves 0.34 Hz even with a pump power as low as 600 μW, while the multiphoton quantum interference visibilities are greater than 95%, and the fidelity is 0.78 ± 0.02. This four-photon tensor-product state could be projected to be a Greenberger–Horne–Zeilinger (GHZ) state with 50% probability and further be used in quantum information applications^33^.

## Results

### Experimental setup

**Fig. 1:**
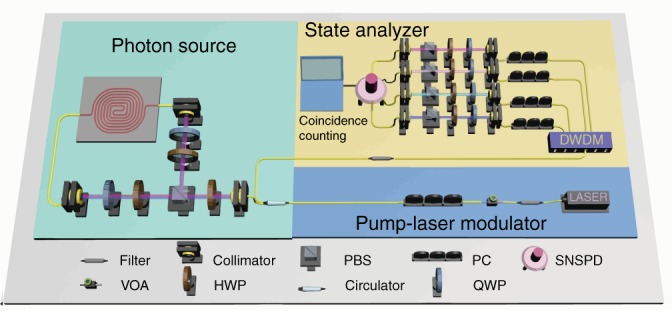
Schematic configuration of our system for the generation and characterization of the multiphoton quantum state with a silicon nanophotonic waveguide. A pulse erbium-doped fiber laser with a repetition rate of 100 MHz was used as the pump light. After a VOA and a prefilter with a bandwidth of 100 GHz, the pump light was input into a Sagnac loop to generate the polarization-encoding quantum state. A postfilter with a bandwidth of 200 GHz was used to block the pump light. A DWDM filter was used to demultiplex photon pairs into the corresponding frequency channels, and a normal architecture for polarization state tomography was used to ascertain the quality of the entangled states. VOA variable optical attenuator, HWP half waveplate, PBS polarization beam splitter, QWP quarter waveplate, PC polarization controller, SNSPD superconducting nanowire single-photon detector

Figure 1 shows the experimental setup for generating multiphoton quantum states with a silicon spiral waveguide. There are three parts included, i.e., the pump–laser modulator, the photon source, and the state analyzer. In the pump–laser modulator, a linearly polarized pulse erbium-doped fiber laser was introduced as the pump source, and had a repetition frequency of 100 MHz and a pulse duration time of 90 fs. A variable optical attenuator was used to precisely modulate the pump power. Then, the pump light went through a 100 GHz bandwidth prefilter, a polarization controller (PC), an optical circulator and was finally coupled into the part of the photon source with horizontal (*H*) polarization by controlling the PC. Here, the coherence time of the pulse laser light was 20 ps after going through this 100 GHz prefilter. In the part of the photon source, we used the configuration with a Sagnac interferometer, which is a popular and self-stabilized scheme for generating polarization-entangled states^12^. The Sagnac interferometer consists of two half-wave plates (HWPs), two quarter-wave plates (QWPs), a polarization beam splitter (PBS), and an ~1 cm-long silicon spiral waveguide with a simple structure as well as a compact footprint (~170 × 170 μm^2^). Before entering the Sagnac interferometer in the part of the photon source, the pump light was modulated to 45° linear polarization [i.e., 50% horizontal (*H*) and 50% vertical (*V*)] by adjusting the HWP carefully. This pump light was then split into the clockwise (*H* polarization) and counterclockwise directions (V-polarization) by a PBS. The HWP and QWP in the Sagnac loop were carefully controlled so that the polarization states of the clockwise and counterclockwise pump pulses were both aligned to the horizontal axis (TE polarization) in the silicon waveguide. In this way, there was only the TE polarization mode propagating along the silicon spiral waveguide on the chip, and no polarization rotation occurred in the light propagation along the silicon spiral waveguides according to the three-dimensional finite-difference time-domain simulation. For the silicon spiral waveguide used here, the propagation loss was approximately 1 dB/cm while the cross section was carefully designed and precisely fabricated to be ~450 × 220 nm^2^ so that near-zero dispersion was achieved in the wavelength band at approximately 1550 nm. Compared with the current multiphoton quantum state results generated with the SFWM process^24,33^, our silicon nanowire source has a broadband near-zero dispersion (see details in Supplementary Fig. S1) and does not have Raman-scattering noise because of the large Raman-scattering frequency shift and the narrow Raman-scattering peak in the silicon^26^. This greatly helps obtain a broadband and uniform SFWM gain spectrum (see details in Supplementary Fig. S1) and, thus, greatly increases the number of photon pairs generated. In addition, with the use of silicon spiral waveguides, one does not need to tune the operation wavelength, which is totally different from the case of using microresonators^24,28^. Furthermore, this waveguide source has a continuous broadband spectrum and short coherence time, which is useful for high-density quantum-key-distribution systems and may enable potential applications in quantum-clock synchronization^26^. In the present case, the clockwise and counterclockwise SFWM processes of TE_p_ + TE_p_ − > TE_s_ + TE_i_ in the silicon spiral waveguide will occur, where TE_p_ is the pump photon in TE polarization and TE_s(i)_ is the signal (idler) photon in TE polarization. These on-chip generated photon pairs in clockwise and counterclockwise directions will further be converted to the states of $\left| {V_{\mathrm{s}}V_i} \right\rangle$ and $\left| {H_{\mathrm{s}}H_i} \right\rangle$, respectively, using the QWP and HWP in the Sagnac loop. Finally, the photon pairs from both directions superpose together by the PBS and output from the Sagnac loop. The biphoton quantum state at the output port of the Sagnac loop is then expressed as follows:

1 $$\left| {\mathrm{\Phi }} \right\rangle = \frac{1}{{\sqrt {1 + \eta ^2} }}\left( {\left| {H_sH_i} \right\rangle + \eta e^{i\delta }\left| {V_sV_i} \right\rangle } \right)$$

where $\left| {V_{\mathrm{s}}V_i} \right\rangle$ and $\left| {H_{\mathrm{s}}H_i} \right\rangle$ are, respectively, the biphoton states generated in the clockwise and counterclockwise directions after the PBS, *η*^*2*^ is the ratio of the *V* and *H* polarization pump powers, and *δ* is the phase difference depending on the birefringence experienced by the signal and idler photons in *HH* and *VV* states. Here, the birefringence is from the other components (except the silicon chip) in the setup since only the TE polarization state is involved on the chip. Grating couplers were used to couple-in the pump light and to couple-out the generated photon pairs. The combination of the HWP and QWP inserted between the PBS and the chip was used to control the optical polarization to maximize the grating coupling efficiency.

Finally, the generated signal and idler photons in the Sagnac loop were output from the circulator port and were then postselected by the part of the state analyzer, as shown in Fig. 1. In this part, a postfilter with a bandwidth of 200 GHz was introduced to block the pump–laser. Then, a 40-channel dense wavelength-division-multiplexing (DWDM) filter with a channel spacing of 100 GHz was introduced to separate the signal and idler photons whose frequencies are equally separated from the central frequency of the pump light. In this way, photon pairs of any combined frequency channels can be selected freely with a broad frequency detuning of about 2 THz from the central pump frequency. The polarization and quantum states of the photon-pairs were unchanged after going through the DWDM filter. Then, the quantum states based on the polarization encoding of the bi- and multiphotons were characterized by the combination of some optical elements and detected by superconducting nanowire single-photon detectors (SCONTEL, the dark count rate DCR = 100 Hz and the detector efficiency *η*_d_ = 85% in the C band).

### Biphoton state

As the first step, the quality of the generated biphoton state was characterized experimentally. Here, the optical pump power was selected to be 120 μW. When the pump light is injected to the Sagnac loop with single direction, the time-correlated photon pairs are generated. Since the photon pairs are frequency multiplexed and generated over all DWDM frequency channels^13^, we selected five pairs of frequency channels for the signal and idler photons to ascertain the effectiveness and stability of our system (Supplementary Table S2). The two-photon coincidences between different combinations of signal and idler channels were measured (Supplementary Fig. S2), and the experimental results showed that the crosstalk is negligible for most frequency channels except some adjacent ones (see details in Supplementary).

By carefully modulating the pump power splitting ratio and the phase difference of the *H* and *V* polarizations, we obtained the maximal polarization-entangled Bell state as follows:

2 $$\left| {\mathrm{\Phi }} \right\rangle = \frac{1}{{\sqrt 2 }}\left( {\left| {H_sH_i} \right\rangle + \left| {V_sV_i} \right\rangle } \right)$$

**Fig. 2:**
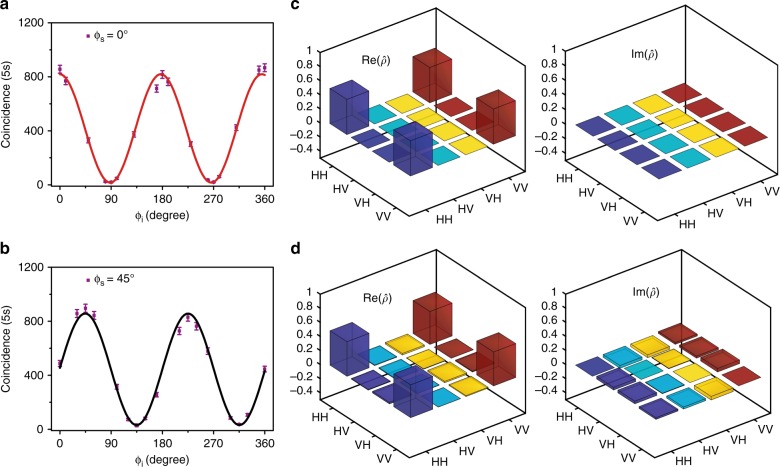
Characterization of biphoton polarization-entangled states. **a** and **b** are twofold coincidences as a function of the idler polarizer angle when the signal polarizer angle was kept at 0° (red) and 45° (black), respectively. The error bar was obtained from the square root of the experimental data. **c** and **d** give the real (Re) and imaginary (Im) parts of the ideal density matrix and the measured density matrix of the biphoton entangled state from frequency channels ±5, respectively. The fidelity was 0.95 ± 0.01, confirming that the generated biphoton quantum state was high quality and very close to the ideal maximally entangled states

To characterize the degree of entanglement, the combinations of QWP, HWP, and PBS after the DWDM filter were used to measure the biphoton interference and to constitute a normal polarization state tomography architecture^34^. The twofold coincidence was recorded by setting the angle of the HWP for the signal channel to 0° or 45° and by modulating the angle of the HWP for the idler channel. The coincidence rate is expected to be proportional to 1 + *V*sin[2*π*(*ϕ*−*ϕ*_c_)/*T*] ^35^, where *V* is the fringe visibility, *ϕ*_c_ is the initial phase, and *T* is the oscillation period. The fringe visibility is defined as *V* = (*d*_max_ − *d*_min_)/(*d*_max_ + *d*_min_), where *d*_min_ and *d*_max_ are respectively the minimum and maximum of the fitted data. Here, we take the signal-idler channels ±5 as an example. The obtained biphoton interference fringes are shown in Fig. 2a, b. Thanks to the high stability and low noise of the whole system, the achieved raw visibilities in the 0° (solid red line) and 45° (solid black line) bases were 96.1 ± 3.2% and 93.0 ± 3.2%, respectively, which are much greater than 70.7%. This result confirms the existence of entanglement and high fidelity of the biphoton state^36^. In this case, the generation rate of the polarization-entangled photon pair is approximately 270 kHz per channel with a pump power of 120 μW, i.e., 0.0027 photon pairs per pulse (see Methods). We obtained an average CAR of approximately 230, which is owing to the ultralow nonlinear noise and the low dark count rate. Such a high CAR leads to a high-raw fidelity for the biphoton source, which is needed to generate the multiphoton entanglement and for further applications of quantum information.

To give a comprehensive characterization, quantum state tomography^37^ was also used. In this way, one can reconstruct the experimental state density matrix by making multiple measurements on identical copies of the relevant quantum state. First, the biphoton quantum state tomography was characterized for signal-idler channels ±5. There are 16 combinations at the measurement basis $\left\{ {H,V,D,R} \right\}^{ \otimes 2}$ acquired to reconstruct the state density matrix, where $$\left| H \right\rangle = \left( {\begin{array}{*{20}{c}} 1 \\ 0 \end{array}} \right),$$ $$\left| V \right\rangle = \left( {\begin{array}{*{20}{c}} 0 \\ 1 \end{array}} \right),$$ $$\left| D \right\rangle = \frac{1}{{\sqrt 2 }}\left( {\begin{array}{*{20}{c}} 1 \\ 1 \end{array}} \right)$$ and $$\left| R \right\rangle = \frac{1}{{\sqrt 2 }}\left( {\begin{array}{*{20}{c}} 1 \\ i \end{array}} \right).$$ Here, the maximum likelihood estimation method was used to reconstruct the density matrix. Figure 2c shows the ideal density matrix $\hat \rho _{ideal}$ of the maximally entangled states (see Eq. (2)) and Fig. 2d shows the measured density matrix $\hat \rho _{mea}$ of the output states. Raw data are used here without subtracting any background or accidental count. The estimated raw fidelity is as high as 0.95 ± 0.01 according to the definition *F* = *Tr*$\left( {\hat \rho _{{\mathrm{mea}}}\hat \rho _{ideal}} \right)$, where *Tr* is the trace. This confirms that the generated biphoton quantum states are of high quality and approach to the ideal maximally entangled states. The error of the fidelity was estimated using 100 Monte Carlo calculations with the Gaussian random data generated from the experimental results. The deviation of the fidelity from unity was mainly due to the nonideal angle rotation of the waveplates. To achieve a high fidelity of greater than 0.9, one should align the wave plate very carefully to minimize the angle error to be less than 2° in the experiment (see the details in Supplementary Fig. S3). It is worth pointing out that the entanglement exists in any signal-idler channels that fulfill the energy conversation condition. For example, we also performed the biphoton quantum state tomography for signal-idler channels ±1 and ±3, and their raw fidelities were 0.94 ± 0.01 and 0.97 ± 0.01, respectively (Supplementary Fig. S4).

### Four-photon state

Since the SFWM spectrum is continuous and the entangled biphoton states were selected by the DWDM filter, multiphoton entangled states can be conveniently generated by multiplexing the biphoton states in different frequency channels (see Supplementary for more details). The multiphoton quantum state generated with our system is expressed as follows:

3 $$\left| {{\mathrm{\Phi }}^{2n}} \right\rangle = \frac{1}{{\sqrt {2^n} }}(\left| {H_{s1}H_{i1}} \right\rangle + \left| {V_{s1}V_{i1}} \right\rangle ) \otimes \cdot \cdot \cdot \otimes (\left| {H_{sn}H_{in}} \right\rangle + \left| {V_{sn}V_{in}} \right\rangle )$$

For example, four-photon quantum states can be obtained by selecting two pairs of signal-idler channels (e.g., ±1 and ±5) with biphoton entangled states given by the following:

4 $$\left| {{\mathrm{\Phi }}_1} \right\rangle = \frac{1}{{\sqrt 2 }}\left( {\left| {H_{s1}H_{i1}} \right\rangle + \left| {V_{s1}V_{i1}} \right\rangle } \right)$$

and

5 $$\left| {{\mathrm{\Phi }}_5} \right\rangle = \frac{1}{{\sqrt 2 }}\left( {\left| {H_{s5}H_{i5}} \right\rangle + \left| {V_{s5}V_{i5}} \right\rangle } \right)$$

respectively. Correspondingly, the obtained four-photon quantum state is given by the following:

6 $$\left| {{\mathrm{\Phi }}^4} \right\rangle = \frac{1}{2}(\left| {H_{s1}H_{i1}} \right\rangle + \left| {V_{s1}V_{i1}} \right\rangle ) \otimes (\left| {H_{s5}H_{i5}} \right\rangle + \left| {V_{s5}V_{n5}} \right\rangle ).$$

**Fig. 3:**
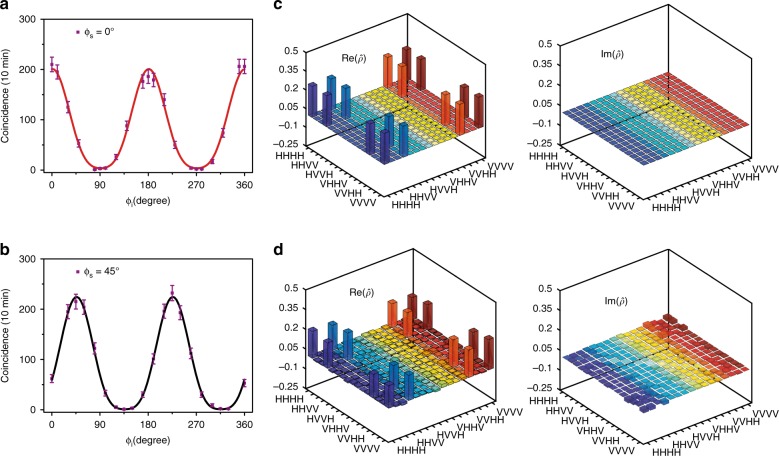
Characterization of four-photon polarization-encoding quantum states. **a** and **b** are fourfold coincidences as a function of the idler polarizer angles when the signal polarizer angles were kept at 0° (red) and 45° (black), respectively. The error bar was obtained from the square root of the experimental data. **c** and **d** give the real (Re) and imaginary (Im) parts of the ideal density matrix and the measured density matrix of the four-photon quantum states, respectively. The fidelity was 0.78 ± 0.02, which is completely satisfactory for further quantum information processing

In our experiment, we obtained a fourfold coincidence rate of 0.34 Hz when the pump power was 600 μW. This result corresponds to a generation rate of 340 kHz as the system and the detection loss was ~15 dB in our case (see Methods). To qualify the quantum state, four-photon quantum interference fringes were measured. Here, the angle of the HWPs for the two signal channels was set as 0° or 45°, while the angle of the HWPs for the two idler channels was modulated from 0° to 180° simultaneously to obtain the interference pattern. Because this four-photon state is the tensor product of two biphoton entangled Bell states, the fourfold coincidence rate is proportional to (1 + sin[2*π*(*ϕ*−*ϕ*_c_)/*T*])^2^ in theory. The four-photon interference patterns measured in this experiment are shown in Fig. 3a, b, respectively. The interference pattern of four-photon states is observed to be in accordance with the theoretical prediction and unfold totally different from the biphoton entangled states. The raw visibilities in the 0° (red) and 45° (black) bases were 96.5 ± 1.5% and 99.1 ± 3.2%, respectively. Such a clear interference pattern and high interference visibilities verify that the technique for constituting on-chip multiphoton quantum states is feasible by multiplexing multiple biphoton entangled Bell states in a silicon nanophotonic waveguide.

For the quantum state tomography of the four-photon quantum states, there were 256 data of the measurement bases $\left\{ {H,V,D,R} \right\}^{ \otimes 4}$ obtained to reconstruct the state density matrix. Figure 3c shows the ideal density matrix of the maximally entangled states (Eq. (6)), and Fig. 3d shows the measured density matrix of the output states from signal-idler channels ±1 and ±5. The fidelity obtained was 0.78 ± 0.02 without subtracting the background and accidental counts. This fidelity is much greater than that (~0.64) realized recently with an integrated optical microcavity on glass^24^ and is fully satisfactory for further quantum information applications.

## Discussion

The multiphoton quantum source demonstrated here has several advantages compared to that in free space. First, the brightness could be very high even with a low-pump power. Our system still has room for further improvement of the collection efficiency, e.g., the efficiency of the chip-fiber coupling and the postfiltering. For example, assuming that the collection efficiency in each channel is improved to 70% (similar to that in free space^9^), the fourfold detected coincidence increases to 42.5 kHz, which is almost one order of magnitude greater than that of free-space four-photon sources^9^ with a pump power of only hundreds of microwatts. As a key component of the collection efficiency, the efficiency of the single grating coupler used in this work is about −5 dB, which, fortunately, can be improved to, e.g., −0.9 dB by introducing a special grating design^38^. This improvement makes a high collection efficiency truly achievable and, thus, offers an opportunity to achieve photonic sources even with 10–10^2^ photons. Furthermore, the present multiphoton quantum source is fully compatible with the on-chip processes of quantum manipulation^39^ as well as quantum detection, which greatly helps constitute large-scale QPICs in the future.

Since the multiphoton source is in the frequency encoding, the single-photon frequency shifting^40^ and the construction of high-fidelity, frequency-based quantum gates^41,42^ can be obtained for high-dimensional quantum information processing. Thus, the present multiphoton source potentially provides an indispensable block for frequency-based quantum information processing. Moreover, one can convert the multiphoton quantum state realized here (Eq. (3)) to the GHZ state through post operation and selection^43^. Our polarization-encoding multiphoton source can also be generalized easily to other degrees of freedom. For example, multiphoton time-bin quantum states can be realized by substituting the Sagnac loop with unbalanced interferometers.

In conclusion, we have experimentally demonstrated the generation of four-photon quantum states with a silicon nanophotonic spiral waveguide. The number of the entangled photons can be increased further with the present system when the collection efficiency is improved. Our multiphoton quantum state source is compatible with contemporary fiber and chip-scale architectures, which makes it very attractive as a scalable and practical platform for future quantum information processing.

## Materials and methods

### System efficiency

The efficiencies in all parts in our system are ascertained as follows. The propagation loss of the silicon nanophotonic waveguides and the coupling loss of the grating couplers were estimated using a cut-back method, which has been used very popularly. Here, we measured the transmissions for short straight waveguides as well as the spiral waveguides on the same chip. The propagation loss and the coupling loss can easily be extracted by linearly fitting the data of the total loss as the waveguide length varies. In the present case, the propagation loss of the silicon spiral waveguide was approximately 1 dB/cm, and the coupling loss for a single grating coupler was approximately 5 dB. The off-chip optical elements in the parts of the photon source as well as the state analyzer had a total loss of 10.3 dB, including a loss of 6 dB from the postfilter and the DWDM filter. For the detectors the efficiency is approximately 85% (−0.7 dB). Therefore, the total losses were ~16dB and ~15 dB for the two-photon and four-photon state measurements, respectively.

### Optical apparatus

We used a pulsed erbium-doped fiber laser whose repetition rate is 100 MHz to generate the pump light. After filtering, the pump light was amplified by an erbium-doped fiber amplifier and filtered again before entering the part of the photon source. For the fiber-chip coupling, the angle of the fiber was set to 10°. Two cascaded off-chip post filters with an extinction ratio of 100 dB were used to remove the pump photons. A DWDM filter, which was used to separate the signal and idler photons, has an extinction ratio of ~30 dB for adjacent channels and ~50 dB for nonadjacent ones. By using superconducting nanowire single-photon detectors, the coincidence detection were recorded, and the electrical pulses were analyzed by a time-correlated single-photon counting system. The coincidence window was set to 0.8 ns for the two-photon state measurements. For the measurements of the four-photon entangled state, the electrical signals were analyzed by UQD-Logic and the coincidence window was set to 1 ns.

## Supplementary Information

supplementary material

## Data Availability

Supplementary information is available in the online version of the paper. Correspondence and requests for materials should be addressed to D.X.D. or X.F.R.

## References

[1] Caspani L (2017). Integrated sources of photon quantum states based on nonlinear optics. Light.

[2] Kimble HJ (2008). The quantum internet. Nature.

[3] Walther P (2005). Experimental one-way quantum computing. Nature.

[4] Humphreys PC (2013). Linear optical quantum computing in a single spatial mode. Phys. Rev. Lett..

[5] Aspuru-Guzik A, Walther P (2012). Photonic quantum simulators. Nat. Phys..

[6] Afek I, Ambar O, Silberberg Y (2010). High-NOON states by mixing quantum and classical light. Science.

[7] Yao XC (2012). Observation of eight-photon entanglement. Nat. Photonics.

[8] Huang YF (2011). Experimental generation of an eight-photon Greenberger–Horne–Zeilinger state. Nat. Commun..

[9] Wang XL (2016). Experimental ten-photon entanglement. Phys. Rev. Lett..

[10] Matsuda N (2012). A monolithically integrated polarization entangled photon pair source on a silicon chip. Sci. Rep..

[11] Tanzilli S (2012). On the genesis and evolution of integrated quantum optics. Laser Photonics Rev..

[12] Takesue H (2008). Generation of polarization entangled photon pairs using silicon wire waveguide. Opt. Express.

[13] Harada KI (2010). Frequency and polarization characteristics of correlated photon-pair generation using a silicon wire waveguide. IEEE J. Sel. Top. Quantum Electron..

[14] Lim HC (2008). Stable source of high quality telecom-band polarization-entangled photon-pairs based on a single, pulse-pumped, short PPLN waveguide. Opt. Express.

[15] Martin A (2010). A polarization entangled photon-pair source based on a type-II PPLN waveguide emitting at a telecom wavelength. New J. Phys..

[16] Suhara T (2009). Quasi-phase-matched waveguide devices for generation of postselection-free polarization-entangled twin photons. IEEE Photonics Technol. Lett..

[17] Takesue H (2007). Entanglement generation using silicon wire waveguide. Appl. Phys. Lett..

[18] Harris NC (2014). Integrated source of spectrally filtered correlated photons for large-scale quantum photonic systems. Phys. Rev. X.

[19] Heeres RW, Kouwenhoven LP, Zwiller V (2013). Quantum interference in plasmonic circuits. Nat. Nanotechnol..

[20] Najafi F (2015). On-chip detection of non-classical light by scalable integration of single-photon detectors. Nat. Commun..

[21] Schuck C (2016). Quantum interference in heterogeneous superconducting-photonic circuits on a silicon chip. Nat. Commun..

[22] Harada KI (2011). Indistinguishable photon pair generation using two independent silicon wire waveguides. New J. Phys..

[23] Spring JB (2017). Chip-based array of near-identical, pure, heralded single-photon sources. Optica.

[24] Reimer C (2016). Generation of multiphoton entangled quantum states by means of integrated frequency combs. Science.

[25] Bogaerts W (2005). Nanophotonic waveguides in silicon-on-insulator fabricated with CMOS technology. J. Light Technol..

[26] Li YH (2017). On-chip multiplexed multiple entanglement sources in a single silicon nanowire. Phys. Rev. Appl..

[27] Silverstone JW (2014). On-chip quantum interference between silicon photon-pair sources. Nat. Photonics.

[28] Silverstone JW (2015). Qubit entanglement between ring-resonator photon-pair sources on a silicon chip. Nat. Commun..

[29] Wang JW (2016). Chip-to-chip quantum photonic interconnect by path-polarization interconversion. Optica.

[30] Wang JW (2017). Experimental quantum Hamiltonian learning. Nat. Phys..

[31] Paesani S (2017). Experimental Bayesian quantum phase estimation on a silicon photonic chip. Phys. Rev. Lett..

[32] Feng LT (2019). On-chip transverse-mode entangled photon pair source. npj Quantum Inf..

[33] McCutcheon W (2016). Experimental verification of multipartite entanglement in quantum networks. Nat. Commun..

[34] Kok P (2007). Linear optical quantum computing with photonic qubits. Rev. Mod. Phys..

[35] Pan JW (2012). Multi-photon entanglement and interferometry. Rev. Mod. Phys..

[36] Braunstein SL, Mann A, Revzen M (1992). Maximal violation of Bell inequalities for mixed states. Phys. Rev. Lett..

[37] James DFV (2001). Measurement of qubits. Phys. Rev. A.

[38] Marchetti R (2017). High-efficiency grating-couplers: demonstration of a new design strategy. Sci. Rep..

[39] Matthews JCF (2009). Manipulation of multiphoton entanglement in waveguide quantum circuits. Nat. Photonics.

[40] Fan LR (2016). Integrated optomechanical single-photon frequency shifter. Nat. Photonics.

[41] Lukens JM, Lougovski P (2017). Frequency-encoded photonic qubits for scalable quantum information processing. Optica.

[42] Lu HH (2018). Electro-optic frequency beam splitters and Tritters for high-fidelity photonic Quantum information processing. Phys. Rev. Lett..

[43] Zhao TM (2014). Entangling different-color photons via time-resolved measurement and active feed forward. Phys. Rev. Lett..

