# Supplementary material for: Generation of multiphoton quantum states on silicon
Source: Light Sci Appl. 2019 May 1;8:41. doi: 10.1038/s41377-019-0153-y (PMC6491612; doi:10.1038/s41377-019-0153-y)
Supplement: Supplementary file 1 — supplementary material [file 41377_2019_153_MOESM1_ESM.docx]

Generation of Multiphoton Quantum States on Silicon

**Ming Zhang1,2*, Lan-Tian Feng3,4*,Zhi-Yuan Zhou3,4, Yang Chen3,4, Hao Wu1,2, Ming Li1,2, Shi-Ming Gao1,2, Guo-Ping Guo3,4, Guang-Can Guo3,4, Dao-Xin Dai****1,2† and Xi-Feng Ren3,4‡**

*­­*1State Key Laboratory for Modern Optical Instrumentation, Centre for Optical and Electromagnetic Research, Zhejiang Provincial Key Laboratory for Sensing Technologies, Zhejiang University, Zijingang Campus, Hangzhou 310058, China.

2Ningbo Research Institute, Zhejiang University, Ningbo 315100, China

3Key Laboratory of Quantum Information, CAS, University of Science and Technology of China, Hefei, Anhui 230026, China.

4Synergetic Innovation Center of Quantum Information & Quantum Physics, University of Science and Technology of China, Hefei, Anhui, 230026, China.

1. **Summary of the generation of the bi/multi-photon quantum state on various χ(3) platforms.**

**Table S1. Typical bi/multi-photon quantum sources on various χ(3) platforms**

| Materials11 | Si | Si | Si | Si | Si | Hydex | Hydex | Si3N4 |
| --- | --- | --- | --- | --- | --- | --- | --- | --- |
| Structures | Nanowire2 | Nanowire  (This work) | Nanowire  (This work) | Ring3 | PhC4 | Ring5 | Ring5 | Ring6 |
| Number of photons | 2 | 2 | 4 | 2 | 2 | 2 | 4 | 2 |
| Nonlinear coefficient (W-1m-1) | 300 | 285 | 285 | ― | 5900 | 0.227 | 0.227 | ― |
| Average pump power (mW) | 1 | 0.12 | 0.6 | 3.3 | 0.6 | 0.6 | 1.5 | 6 |
| Collected photon bandwidth (GHz) | 18 | 50 | 50 | 13 | 12.5 | 0.8 | 0.8 | 0.09 |
| Brightness (pairs s-1) | 40kHz | 270 kHz | 340kHz | 14MHz | ― | 302kHz | 135kHz | 35MHz |
| coincidence–to-accidental ratio | 42 | 230 | ― | 45 | ~5 | ― | ― | ― |
| Raw visibilities of quantum interference | ― | 93.0±3.2% | 96.5±1.5% | 89.3±2.6% | 74.1±4.8% | 82.4% | 89% | ~90% |
| Fidelity | 0.91±0.02 | 0.95±0.01 (raw) | 0.78±0.02 | ― | ― | 0.96 (net) | 0.64 | ― |

While the quantum sources using bulk crystals is quite mature, it is desired to develop chip-scale quantum technologies using compact photonic integrated circuits1. This will reduce cost, footprint, and energy consumption and greatly increase reliability.

Table S1 gives a summary for the state-of-the-art for the generation of the bi/multi-photon quantum sources on various χ(3) platforms. For photon-pair sources, we just compare the works for realizing the entangled states preparation. We classify these integrated sources according to whether they are based on waveguides or cavities. The source brightness and the coincidence-to-accidental ratio (CAR) are the two most important parameters for characterizing the quality of a photon pair source. The source brightness could be increased by adding the pump power, however, the nonlinear noise and multiphoton terms also increase, which are harmful for improving the CAR. It is proved that one can achieve high brightness without decreasing the CAR when using microring resonators, pulsed laser pump and low-dark count detectors. In our experiment, we achieved the biphoton entangled source with the highest CAR among all the generated biphoton entangled sources. This is helpful to prepare the four-photon quantum state with a high quality.

In order to give a quantitative representation for the relationship between the source brightness (the net coincidence rate *C*) and the CAR, we introduced the following series of equations8. The net coincidence rate *C* is proportional to the photon-pair generation rate *r* of the spontaneously four-wave mixing (SFWM) process, and one has

$$\begin{matrix} C=\sigma\eta_{\alpha}^{2}\eta^{2}\eta_{s}\eta_{i}r, \\ r=\Delta\upsilon\left(\gamma P_{0}L_{eff}\right)^{2}\text{sinc}^{2}\left[\beta_{2}\left(2\pi\nu\right)^{2}L/2+\gamma P_{0}L\right], \end{matrix}$$

where ∆*υ* is the bandwidth of the pair-photon channel, *γ* is the nonlinear coefficient estimated based on the nonlinear refractive index, *P*0 is the pump energy, *v* is the signal frequency shift from the pump channel, *L* is the waveguide length, *L*eff is the effective waveguide length taking into account the propagation loss *α* (i.e., *L*eff=[1−exp(−*αL*)]/*α*), *β*2 is the second-order dispersion parameter, *ηα* is a parameter taking into account the pair-photon loss in the waveguide, *η* is the fiber-chip coupling efficiency, *ηs* and *ηi* are respectively the efficiencies including post-processing and detection efficiencies for the signal and the idler photons. The single-channel counts *Ns*(*i*) are related with the photon-pair generation nonlinear process, the noise-photon generation nonlinear process and the detectors’ dark count. One has

$$\begin{matrix} N_{s}=\sigma\eta\eta_{s}\left(\eta_{\alpha}r+r_{ns}\right)+d_{s}, \\ N_{i}=\sigma\eta\eta_{i}\left(\eta_{\alpha}r+r_{ni}\right)+d_{i}, \end{matrix}$$

where *rns* and *rni* are respectively the noise-photon generation rates for the signal and the idler photons, *d*s and *d*i are respectively the rates of detectors' dark count for the signal and the idler photons, σ is the duty cycle parameter (*σ*=*τB*, where *τ* is the pulse width and *B* is the pulse repetition rate). One has *σ*=1 for the CW operation. The accidental rate *A* could be obtained from the single-channel counts *Ns*(*i*). One has *A*=*NsNit* for the CW operation in the time window of *t* for the coincidence measurement (i.e., the FWHM of the coincidence peak). For the pulsed-laser operation, one has *A*=*N*0*N*1/*B*. Finally, the CAR is the coincidence to the accidental ratio *A*, i.e., CAR=*C*/*A*. Note that in real experiment the measured coincidence *C*mea is the sum of the net coincidence rate *C* and the accidental ratio *A*. Thus, one has

CAR=(*C*mea−*A*)/*A*.

We can conclude that the source brightness mainly determined by the pump intensity and effective waveguide length according to Eq. (1). It can be seen that the source brightness as a function of the waveguide-coupled peak pump power *P*0 shows quadratic pattern. For the waveguide length, there is little point in using waveguides significantly longer than a distance 1/*α*, since the intensity is too weak to contribute to the photon flux and *L*eff is close to its maximum 1/*α*. The CAR as a function of the pump power usually rises first and then falls down, as shown in the Fig. 2b and 3b of Ref. [9]. At the low pump power regime, the CAR is mainly influenced by the dark count of the detectors. In contrast, at the high power regime the multiphoton process is the reason for the CAR reduction10.

1. **Theory for the multiphoton quantum state preparation**

In our experiment, the interaction Hamiltonian for the entangled photon pair source by SFWM process in each direction of the Sagnac loop is described as

,

where $\chi$ is proportional to the third-order nonlinear susceptibility and the pump power, $a_{xi}^{\dagger}\text{ }\left(a_{xi}\right)$ and $a_{xs}^{\dagger}\text{ }\left(a_{xs}\right)$ are the creation (annihilation) operators for different idler and signal photons emitted from the source, respectively. Here, it is reasonably assumed that the pump light is strong and can be treated as a classical oscillator. The time evolution of the quantum state from the output port of the Sagnac loop is given by

,

where, $\hat{H}$ represents the total Hamiltonian defined as $\hat{H}=\left(\hat{H}_{H}+\hat{H}_{V}\right)/\sqrt{2}$, and $|mn\rangle_{is}$ is the number states, in which *m* and *n* are the numbers of idler and signal photons. By using the disentangling theorem11, Eq. (4) can be re-written as

$\begin{matrix} |\Psi\rangle=\frac{1}{C^{2}}e^{\frac{1}{\sqrt{2}}\Gamma\left(a_{Hi}^{\dagger}a_{Hs}^{\dagger}+a_{Vi}^{\dagger}a_{Vs}^{\dagger}\right)}|0\rangle_{i}|0\rangle_{s} \\ \text{ }=\frac{1}{C^{2}}\left(1+\hat{H}'+\frac{1}{2!}\hat{H}'^{2}+\frac{1}{3!}\hat{H}'^{3}+\cdots\right)|0\rangle_{i}|0\rangle_{s} \end{matrix}$,

where $\hat{H}'=\Gamma\left(a_{Hi}^{\dagger}a_{Hs}^{\dagger}+a_{Vi}^{\dagger}a_{Vs}^{\dagger}\right)/\sqrt{2}$, $C=\cosh\left(\chi t\right)\approx1$ and $\Gamma=\tanh\left(\chi t\right)$. Therefore, the two-photon entangled state could be expressed from the second term as

$|\Phi\rangle=\frac{1}{\sqrt{2}}\left(|H_{i}H_{s}\rangle+|V_{i}V_{s}\rangle\right)$.

For the four-photon quantum state constituting by the tensor product of two biphoton entangled Bell states, the interaction Hamiltonian could be expressed as

$\hat{H}=\frac{1}{\sqrt{2}}\left(\hat{H}_{i1s1}+\hat{H}_{i2s2}\right)$,

where $\hat{H}_{i1s1}=\left(\hat{H}_{H}+\hat{H}_{V}\right)_{i1s1}/\sqrt{2}$, $\hat{H}_{i2s2}=\left(\hat{H}_{H}+\hat{H}_{V}\right)_{i2s2}/\sqrt{2}$, $i_{1}$, $s_{1}$, $i_{2}$ and $s_{2}$ represent the four photons in different frequencies, respectively. The time evolution of the quantum state can be expressed as follows

,

where $\hat{H}'=\Gamma\left(a_{Hi1}^{\dagger}a_{Hs1}^{\dagger}+a_{Vi1}^{\dagger}a_{Vs1}^{\dagger}\right)/\sqrt{2}$ and $\hat{H}''=\Gamma\left(a_{Hi2}^{\dagger}a_{Hs2}^{\dagger}+a_{Vi2}^{\dagger}a_{Vs2}^{\dagger}\right)/\sqrt{2}$. The post-selection measurement extracts the four-photon terms $|1\rangle_{i1}|1\rangle_{s1}|1\rangle_{i2}|1\rangle_{s2}$ and the four-photon state could be expressed as

$|\Phi\rangle=\frac{1}{2}\left(|H_{i1}H_{s1}\rangle+|V_{i1}V_{s1}\rangle\right)\otimes\left(|H_{i2}H_{s2}\rangle+|V_{i2}V_{s2}\rangle\right)$.

This four-photon quantum state is proven in our experiment and photonic states with more photon numbers could be achieved with the same process.

1. **Wavelengths in each DWDM channel**

In our experiment, the DWDM filter has 40 channels and the channel spacing is 100 GHz, such that one can select the combinations of photon pairs within a frequency detuning about 2 THz. Five pairs of frequency-channels to ascertain the effectiveness and stability of our system. The corresponding wavelengths for the signal-idler channels are shown in Table S2.

**Table S2**. **Wavelengths of the standard ITU grids for the** selected five **signal and idler photons**.

| Pair number | DWDM channel | Wavelength (nm) |
| --- | --- | --- |
| Signal 5, idler -5 | C19, C49 | 1562.23, 1538.19 |
| Signal 4, idler -4 | C20, C48 | 1561.42, 1538.98 |
| Signal 3, idler -3 | C21, C47 | 1560.61, 1539.77 |
| Signal 2, idler -2 | C22, C46 | 1559.79, 1540.56 |
| Signal 1, idler -1 | C23, C45 | 1558.98, 1541.35 |
| Pump | C34 | 1550.12 |

1. **Dispersion and normalized SFWM gain in silicon nanowire waveguides**

It is crucial to tailor the dispersion of a silicon nanowire waveguide in order to increase the efficiency of SFWM processes12, and thus one should optimize optical confinement in the waveguide. In our case, the dispersion curves of silicon nanowire waveguides with different core widths *w*co were calculated by using the numerical tool from Lumerical MODE Solution. Figure S1 shows the calculated group velocity dispersion for three waveguides with wco=440, 450, and 460 nm. It can be seen that the dispersion is near zero at 1550 nm when the core width wco is around 450 nm. In addition, it is still easy to achieve near-zero dispersion even when there is some fabrication error. The normalized SFWM gain is also calculated and shown in Fig. S113. It can be seen that the normalized SFWM gain spectrum is wavelength-insensitive and the bandwidth is as large as dozens of THz. This indicates that the present silicon platform is superior for building on-chip quantum photonic devices.


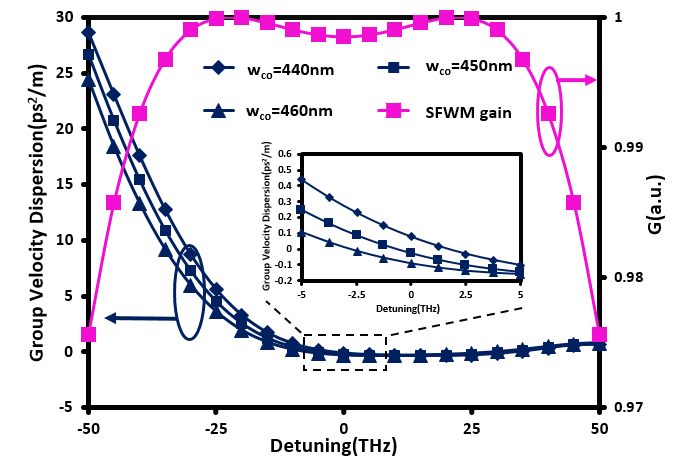


**Fig. S1: Calculated dispersion curves for silicon nanowire waveguides with different core widths and the normalized SFWM gain for the case of *w*co=450 nm with pump light at 1550.12 nm.** Inset: zoom-in view.

1. **Two-photon coincidences between different frequency-channels**

The entangled photon pairs can be generated over all DWDM frequency-channels because of the strong SFWM process in the broad band. As an example, we selected five pairs of signal-idler channels to ascertain the precision and stability of our system (Supplementary Table 1). Fig. S2 showsthe recorded two-photon coincidences between different combinations of signal-idler channels. Here the pump light with a power of 120 μW was injected into the Sagnac loop. It can be seen that the crosstalk is negligible for most frequency-channels except the pairs (1,-2), (2,-3), (3,-4) & (4,-5). The crosstalk might mainly come from some wavelength misalignment between the pump wavelength (defined by the pre-filter in the setup) and the central-channel wavelength of the DWDM filter. This crosstalk can be avoided when selecting nonadjacent frequency-channels, as demonstrated in the main text.


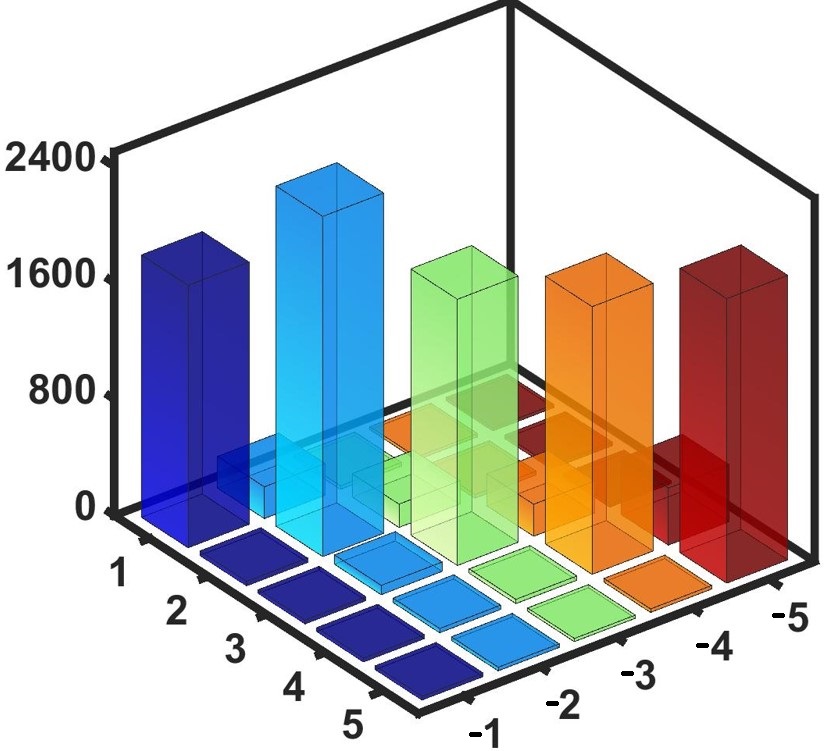


**Fig. S2: Experiment results of two-photon coincidences for the selected five pairs of signal-idler channels.** Here the pump power injected to the Sagnac loop is 120 μW. The two-photon coincidences were measured for the selected five pairs of signal-idler channels.

1. **The impact of the angle error on the tomography fidelity**


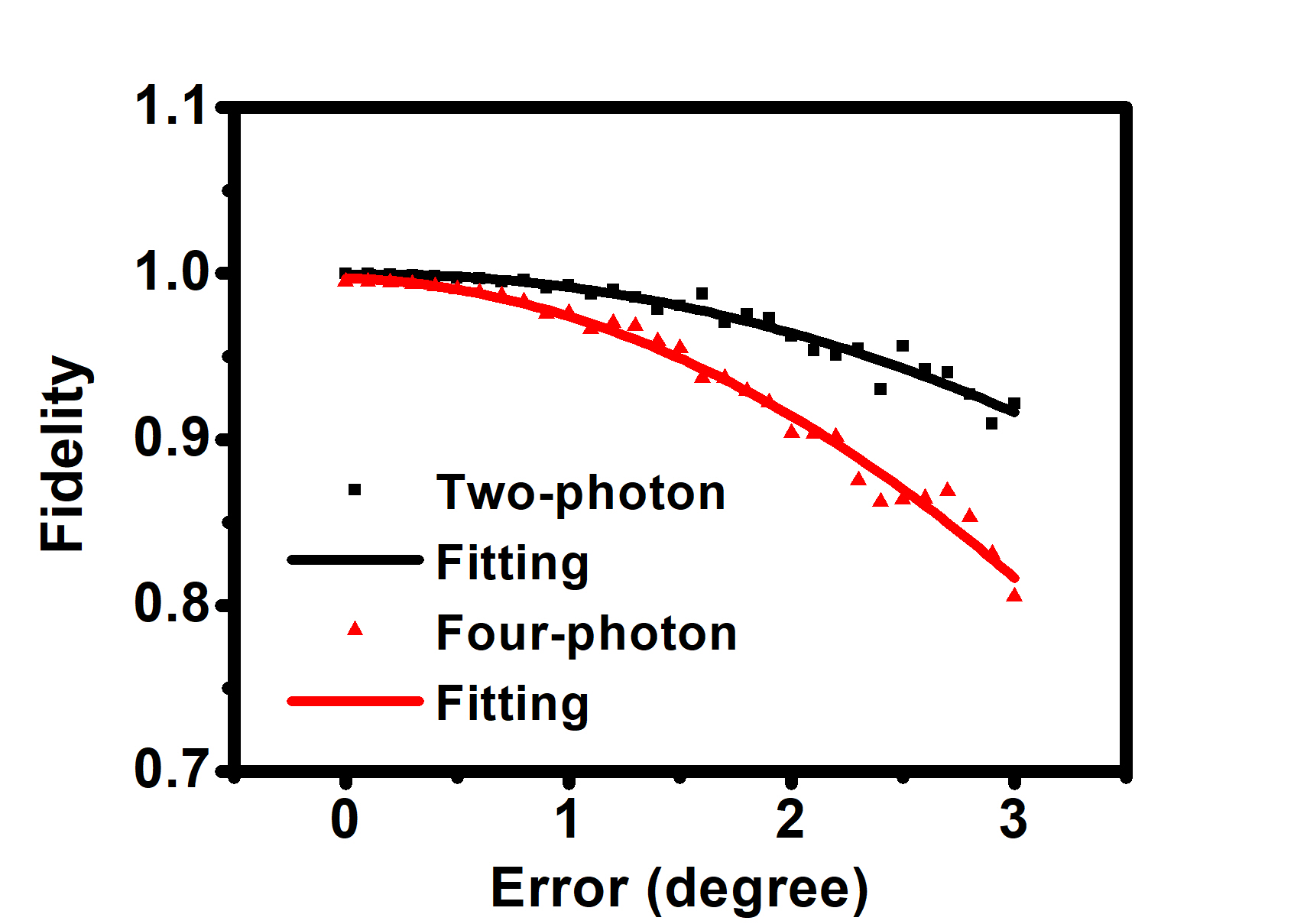


**Fig. S3**. **Impact of the angle error on the tomography fidelity for the two-photon state (black squares) and the four-photon state (red triangles).** The four-photon state is more sensitive than the two-photon state. The data are the average result of ten times of calculations. The curves are the polynomial fitting of the data.

As it is well known, the angle-rotation of the waveplates is not perfect in reality, which introduces some impact on the tomography fidelity. In order to estimate this impact, we calculate the tomography fidelity for the four-photon state and the two-photon state by assuming that there was an angle error *δ* introduced in the measurement, as shown in Fig. S3. It can be seen that the four-photon state is more sensitive than the two-photon state. In order to achieve a high fidelity above 0.9, one should align the wave-plate very carefully to minimize the angle error to be less than 2° in the experiment.

1. **Two-photon quantum state tomography of other signal-idler channels**

The generated photon pairs are frequency multiplexed and highly entangled for any selected signal-idler channels which fulfills the energy conversation condition. To verify this, we also performed the two-photon quantum state tomography for signal-idler channels ±1 and ±3, and the measured raw fidelities for them are 0.94±0.01 and 0.97±0.01, respectively.


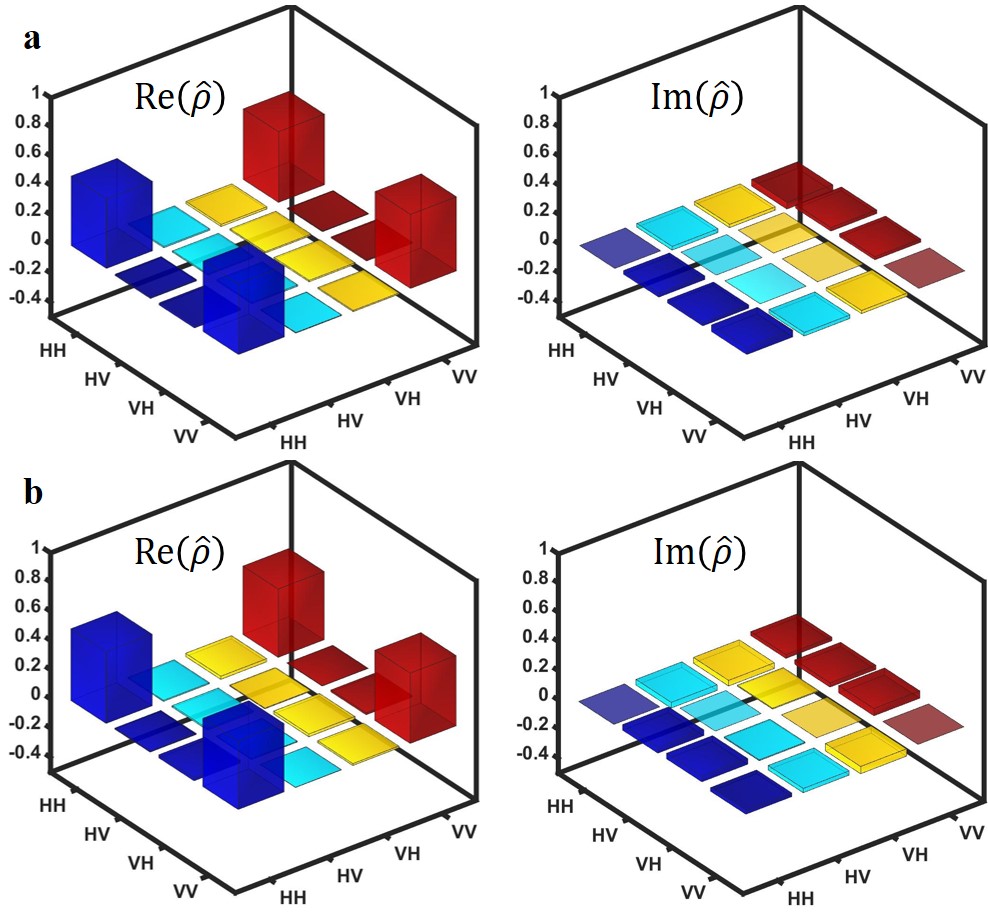


**Fig. S4**. **Quantum state characterization through state tomography.** A quantum state can be fully described by its density matrix $\hat{\rho}$. The real (Re) and imaginary (Im) parts of the measured density matrices of two-photon qubit state in signal-idler channels ±1 and ±3 are shown in **a** and **b**, respectively. The corresponding estimated fidelities are 0.94±0.01 and 0.97±0.01, respectively.


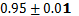


**References**

1. Caspani, L. *et al*. Integrated sources of photon quantum states based on nonlinear optics. *Light Sci. Appl*. **6,** e17100 (2017).
2. Matsuda, N. *et al.* A monolithically integrated polarization entangled photon pair source on a silicon chip. *Sci. Rep*. **2,** 817 (2012).
3. Grassani, D. *et al*. Micrometer-scale integrated silicon source of time-energy entangled photons. *Optica* **2,** 88–94(2015).
4. Takesue, H., Matsuda, N., Kuramochi, E. & Notomi, M. Entangled photons from on-chip slow light. *Sci. Rep.* **4,** 3913(2014).
5. Reimer, C. *et al*. Generation of multi-photon entangled quantum states by means of integrated frequency combs. *Science* **351,** 1176-1180 (2016).
6. Ramelow, S. *et al*. Silicon-nitride platform for narrowband entangled photon generation. arxiv:1508.04358.
7. Moss, DJ. *et al*. New CMOS-compatible platforms based on silicon nitride and Hydex for nonlinear optics. *Nat Photonics* **7**, 597–607 (2013).
8. Xiong, C. *et al*. Generation of correlated photon pairs in a chalcogenide As2S3 waveguide. *Appl. Phys. Lett.* **98,** 051101 (2011).
9. Li, Y. H. *et al*. On-Chip Multiplexed Multiple Entanglement Sources in a Single Silicon Nanowire. *Phys. Rev. Appl.* **7,** 064005 (2017).
10. Harada, K. *et al*. Frequency and polarization characteristics of correlated photon-pair generation using a silicon wire waveguide, *IEEE J. Sel. Top. Quantum Electron.* **16,** 325 (2010).
11. Harada, K. I. *et al.* Indistinguishable photon pair generation using two independent silicon wire waveguides. *New J. of Phys.* **13,** 065005 (2011).
12. Turner, A C. *et al*. Tailored anomalous group-velocity dispersion in silicon channel waveguides. *Optics express.* **14(10),** 4357-4362 (2006).
13. Brainis, E. Four-photon scattering in birefringent fibers. *Physical Review A*, **79(2),** 023840 (2009).
